# The effects of thawing on the plasma metabolome: evaluating differences between thawed plasma and multi-organ samples

**DOI:** 10.1007/s11306-017-1196-9

**Published:** 2017-04-17

**Authors:** Frida Torell, Kate Bennett, Stefan Rännar, Katrin Lundstedt-Enkel, Torbjörn Lundstedt, Johan Trygg

**Affiliations:** 1 Department of Chemistry, Computational Life Science Cluster (CLiC), Umeå University, Umeå, Sweden; 2 Karlsruhe Institute of Technology, Karlsruhe, Germany; 3 AcureOmics AB, Umeå, Sweden; 4 Department of Organismal Biology, Uppsala University, Uppsala, Sweden; 5 Department of Pharmaceutical Biosciences, Uppsala University, Uppsala, Sweden

**Keywords:** Mouse, Metabolomics, Plasma, Multi-organ, Freeze–thaw cycle, OPLS-DA

## Abstract

**Introduction:**

Post-collection handling, storage and transportation can affect the quality of blood samples. Pre-analytical biases can easily be introduced and can jeopardize accurate profiling of the plasma metabolome. Consequently, a mouse study must be carefully planned in order to avoid any kind of bias that can be introduced, in order not to compromise the outcome of the study. The storage and shipment of the samples should be made in such a way that the freeze–thaw cycles are kept to a minimum. In order to keep the latent effects on the stability of the blood metabolome to a minimum it is essential to study the effect that the post-collection and pre-analytical error have on the metabolome.

**Objectives:**

The aim of this study was to investigate the effects of thawing on the metabolic profiles of different sample types.

**Methods:**

In the present study, a metabolomics approach was utilized to obtain a thawing profile of plasma samples obtained on three different days of experiment. The plasma samples were collected from the tail on day 1 and 3, while retro-orbital sampling was used on day 5. The samples were analysed using gas chromatography time-of-flight mass spectrometry (GC TOF-MS).

**Results:**

The thawed plasma samples were found to be characterized by higher levels of amino acids, fatty acids, glycerol metabolites and purine and pyrimidine metabolites as a result of protein degradation, cell degradation and increased phospholipase activity. The consensus profile was thereafter compared to the previously published study comparing thawing profiles of tissue samples from gut, kidney, liver, muscle and pancreas.

**Conclusions:**

The comparison between thawed organ samples and thawed plasma samples indicate that the organ samples are more sensitive to thawing, however thawing still affected all investigated sample types.

**Electronic supplementary material:**

The online version of this article (doi:10.1007/s11306-017-1196-9) contains supplementary material, which is available to authorized users.

## Introduction

Metabolomic studies involve several steps starting from the hypothesis or study design and resulting in a biological interpretation (Brown et al. 2005; Krastanov 2010). It is essential to use a carefully planned experimental design, since metabolomics studies are complicated by the high temporal and spatial variability that the metabolites demonstrate e.g. physical properties, chemical properties and concentration range (Korn et al. 2007). The experiments must rest on a well thought-out foundation, making the design unfold in a proper fashion. The pipeline for a metabolomics approach often involves experimental planning, sampling, storage, pre-treatment, chemical analysis, data processing, multivariate data analysis, validation, and biological interpretation. The outcome of the study as well as the quality of the results is highly dependent on how well each step of the pipeline is executed. In order to obtain reliable and reproducible results, standardised protocols have been proposed (Lindon et al. 2005). However, steps that are often overlooked include the experimental planning, sampling and transportation of samples and the effects that these have on the final results and the biological interpretation.

Blood samples are commonly used in metabolomics studies. Blood samples mainly contains endogenous metabolites, but also food and drug metabolites, so called exogenous metabolites (Bouatra et al. 2013). Nonetheless, the sampling can easily affect the metabolome. The metabolite concentration in blood can be affected by the time the sample spends in room temperature, storage conditions as the time left to stand at room temperature prior to centrifugation (Yin et al. 2015). In order to overcome these problems it is essential to follow a standard operating procedure (SOP), such as the one utilized in the present study (Holland et al. 2005).

The stability and reproducibility of metabolite measurements as a response to shipment has previously been investigated with a focus on a small number of metabolites, in whole blood. The investigated metabolites include e.g. amino acids (Davis et al. 2009), cholesterol (Giltay et al. 2003; Clark et al. 2003; Key et al. 1996), fatty acids (Eijsden et al. 2005), glucose (Bruns and Knowler 2009), lipids (Key et al. 1996; Eijsden et al. 2005) and vitamins (Key et al. 1996). In addition, a study has been performed, addressing the reproducibility of targeted metabolomics using human plasma and serum samples from different time-points (Breier et al. 2014). The focus of these studies have been to identify alterations associated with repeated freeze–thaw cycles as well as different shipment conditions.

In a previous study the authors studied metabolic alterations associated with thawing of multiple organ samples, including gut, kidney, liver, muscle and pancreas (Torell et al. 2015). It was found that the metabolite levels in tissue samples reacted highly similar to thawing. In the present study, the authors present the metabolic changes observed as a response to thawing of plasma samples obtained from the same mice on three different days of experiments. Since the results of thawing studies have previously been inconclusive, more work is needed in this area. The aim of this study was to investigate differences in thawing sensitivity and metabolite stability, between different sample types (i.e. gut, kidney, liver, muscle, pancreas and plasma). This was achieved by performing a comparative study between the consensus plasma profile and the thawing effects on multi-organ samples. Initially, the metabolic profile associated with changes observed in plasma samples, as a result of thawing, was obtained. Thereafter, the differences and similarities between plasma and organ samples, in response to thawing was evaluated.

## Materials and methods

### Samples

Eight-month-old wild-type male mice of the mixed background 129sv x C57Bl/6N (n = 15) were individually placed in metabolic cages (Metabolic cage for mice, Tecniplast™, UK), with access to food [ref A04-10 in powder, Scientific animal food & engineering (SAFE), France] and water. The animals were kept 5 days in the metabolic cages, 2 days as an adaptation period and 3 days of experiment. Each experimental day, animals were weighed. Food and water intake, urine volume and fecal weight for each mouse were recorded. Urine and feces were collected every 24 h, and frozen at −80 °C for storage until analysis. After 4 h fasting, 50 µL of blood from the tail was recovered in heparin tubes (Microvette CB300 LH, Sarstedt, Germany). Plasma was obtained from the blood sample by centrifugation (2000 rpm, 5 min at 20 °C), and frozen at −80 °C. The fifth day of experiment, animals were anesthetized with a ketamine and xylazine solution by intraperitoneal injection, a blood sample was obtained by retro-orbital bleeding performed with a heparinized Pasteur pipette, and plasma was prepared as described above. Animals were killed by cervical dislocation. Animal experiments were conducted in accordance with French and European ethical legal guidelines and the local ethical committee for animal care (Comité d’éthique en expérimentation animale Charles Darwin No. 5, approval number No. 01508.01) specifically approved this study.

All of the samples were placed on blocks of dry ice (3 kg) in two separate, sealed, styrofoam boxes. One box was shipped for 24 h, in which all the samples arrived to the destination frozen. In contrast, the second box was shipped for a total of 72 h, after which all samples arrived to the destination thawed. The organ samples were divided into two separate groups, based on the shipment time. No significant changes in metabolite concentration have been observed during 24 h shipment (Breier et al. 2014). Therefore, the groups have been referred to as ‘frozen’ and ‘thawed’ (n1 = 8 × 3 and n2 = 7 × 3, respectively). All samples were stored at −80 °C until metabolic profiling analysis.

### Metabolomic analysis

Metabolites were extracted from 50 µL plasma aliquots according to the method described by A et al. (2005). The samples were then analysed using gas chromatography time-of-flight mass spectrometry (GC TOF-MS) and detected metabolites were identified and quantified as described by Jonsson et al. (2005) Complete method description is available as supplementary data. As a means of quality control to account for any analytical variation and to test the reproducibility of the method, several samples were performed in triplicates. In order to account for any instrumental variation and to monitor run order effects, the triplicates were separated throughout the run list during the GC–MS analysis.

### Data analysis

Variance was checked by means of F-test. p values were calculated by applying Student’s *t* test for samples of equal variance or unequal variance, depending on the result of the F-test. The *t* tests were then corrected for multiple testing using Benjamini–Hochberg FDR method (Benjamini and Hochberg 1995). The false discovery rate was set to be 5%.

Person correlation coefficients were calculated based on the p(corr)-loading values from OPLS-DA frozen versus thawed models to measure how well the thawing profiles correlated. The results of Pearson correlation range between −1 and 1, where high correlation is indicated if the absolute value is between ± 0.5 to ± 1, medium correlation range between ± 0.3 to ± 0.5 and values between 0 and ± 0.3 indicate low correlation.

Multivariate analysis was performed on unit variance scaled data using SIMCA, version 14.0 (Umetrics AB, Umeå, Sweden). Multivariate data analysis was based on principal component analysis (PCA) (Jackson 1991), orthogonal projection to latent structures (OPLS) (Trygg and Wold 2002) and orthogonal projection to latent structures discriminant analysis (OPLS-DA) (Bylesjö et al. 2006). Two components were calculated for all types of models. As a part of quality control the data was examined by means of run order control and quality control of triplicates. The triplicates and duplicates were in close proximity, in the same quadrant in the PCA score scatter plot, i.e. displaying similar characteristics. Therefore, an average of each of the replicates was used for further data analysis.

PCA is an unsupervised multivariate technique where the variation is summarized into a smaller number of latent variables, so called principal components. The latent variables are used to visualize the observations (samples). Thereby, the relationship between observations that are characterized by many variables can be visualized in low dimensional plots (Wold et al. 1987).

OPLS is a supervised method which finds the variation in X (the metabolic data) that is correlated to Y (in our study, the run order). OPLS summarizes the systematic variation in X that is correlated to Y in one component, called the predictive component (t1).Simultaneously it also finds the systematic variation in X that is uncorrelated to Y, and places it in other components, called orthogonal components (to) (Trygg and Wold 2002). An OPLS model was calculated in order to assess the size of the run order effects.

OPLS-DA is an extension of OPLS. OPLS-DA is a supervised method which finds the variation in X (the metabolic data) that is correlated to Y (in our study, dummy variables representing frozen and thawed samples). In case of a two-class problem, OPLS-DA summarizes all the relevant variation in one predictive component (t1), while the systematic variation in X that is uncorrelated to Y, is placed in one orthogonal component (to) (Bylesjö et al. 2006). Variable significance was determined by interpreting the correlation scaled loading values (p(corr)), from the OPLS-DA model, together with the 95% confidence intervals calculated using jack-knifing (derived by seven-fold internal cross-validation). ANOVA of the cross-validated residuals (CV-ANOVA) was used to assess the significance of the OPLS-DA models (Eriksson et al. 2008).

The shared and unique structure (SUS) plot is produced by plotting the predictive components (p(corr)) from two different models, against one another. The resulting plot displays both shared and unique features for these two models. The shared features will be found in the diagonal and unique features in the areas close to the x- and y-axis. Hence, the SUS-plot can be used to find metabolites that vary in the same direction in both models, as well as metabolites that differ between the two models (Eriksson et al. 2013).

## Results

### Quality control

The metabolomic analysis resulted in the detection of 159 metabolites, out of which 46 could be positively identified using our in-house libraries. The multivariate data analysis was performed using the identified metabolites only. Initially, the samples that had been analysed in triplicates were evaluated using PCA. The multivariate data analysis found each set of triplicates to be clustered together in the score plot, confirming high reproducibility of the method. The run order effects were analysed by creating an OPLS model using the run order as the response variable. The run order analysis showed that no severe run order effects could be found (the predictive component was 0.07).

### Frozen versus thawed plasma samples

The eight samples from each time point that arrived frozen were initially compared to the seven samples from each time point that arrived thawed. In order to get an overview of these samples a PCA model was calculated, which resulted in a two component model where R^2^X = 0.43. The PCA score plot can be found in the supplementary data.

On the fifth day, the mice were given anaesthesia. The animals were anaesthetised with Ketamine/Xylazine, which can have profound effects on the global metabolic profiles (Overmyer et al. 2015). It should be noted that on day 1 and day 3, the blood was drawn from the tail which resulted in a mix of venous and arterial blood whereas the sinus retro-orbital sampling procedure used on day 5 gave only venous blood. This may have introduced a difference in metabolic composition between the different sampling days (Hoff and Rlagt 2000). Therefore, the samples from the fifth day were investigated separately. This was performed in order to assess the similarities and dissimilarities between the thawing of plasma samples where blood was drawn from the tail, without anaesthesia, and plasma samples drawn from the eye, where anaesthesia was used.

#### Frozen versus thawed plasma samples from day 1 and 3

**Fig. 1:**
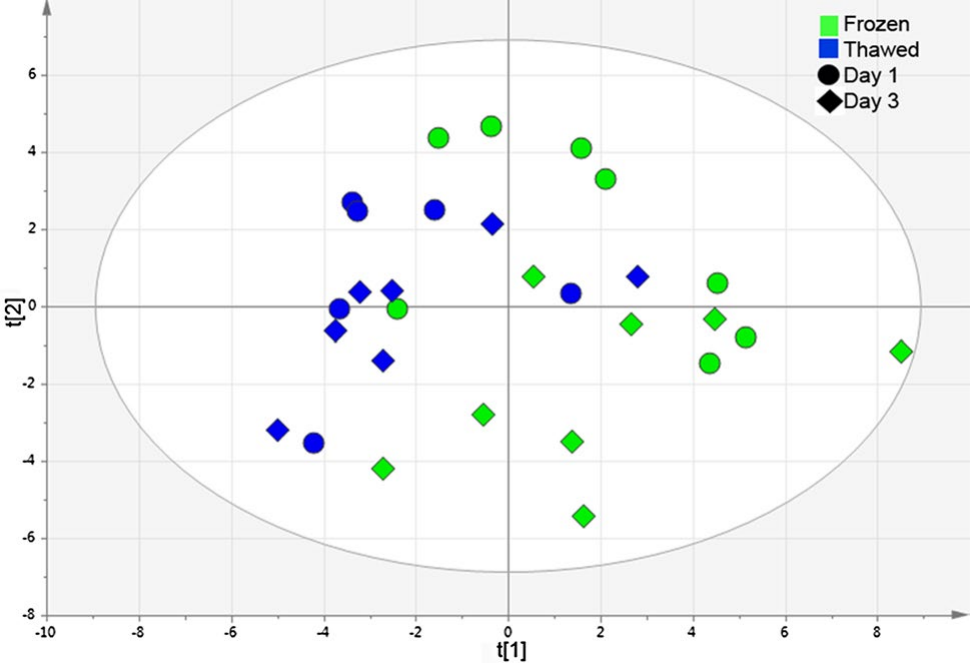
PCA score plot with frozen and thawed samples from day 1 and day 3. The two components explained 25 and 15% of the variation, respectively. The first component explained approximately the thawed and frozen plasma samples. There was a tendency for the thawed samples to end up on the left-hand side in the score plot, while the frozen samples had a tendency to end up on the right-hand side. The *blue* and *green* spots represent the thawed samples (T) and the frozen samples (F), respectively. Samples from day 1 are represented by *circles* and samples from day 3 are represented by *diamonds*

In order to obtain an overview of the thawed and frozen samples from day 1 and 3, a new PCA model was calculated for the samples from day 1 and day 3. The two components explained 25 and 15% of the variation, respectively. The first component explained approximately the thawed and frozen plasma samples from day 1 and 3. The score scatterplot can be seen in Fig. 1.

Thereafter, OPLS-DA models were calculated in order to compare thawed and frozen samples from day 1 and day 3, see Table 1.

**Table 1:** OPLS-DA models for frozen versus thawed plasma samples

Name	Type	A	N	R^2^X	R^2^Y	Q^2^Y	p value^a^
Frozen versus thawed day 1	OPLS-DA	1 + 1 + 0	15	0.48	0.92	0.70	0.01
Frozen versus thawed day 3	OPLS-DA	1 + 1 + 0	15	0.46	0.95	0.80	0.002

Eight samples from each time-point arrived frozen, while seven from each time-point arrived thawed

*A* number of components, *N* number of samples that the model is based on, *R* ^2^ “goodness of fit” parameter that shows how well the model describes the variation in the data. R^2^X, R^2^Y are the cumulative variations explained in the metabolite and class-variable data respectively, *Q* ^2^ *Y* “goodness of prediction” parameter and is the cross-validated prediction estimate of class separation that shows how well samples are predicted by the model

^a^p values were obtained using CV-ANOVA in SIMCA 14.0

**Fig. 2:**
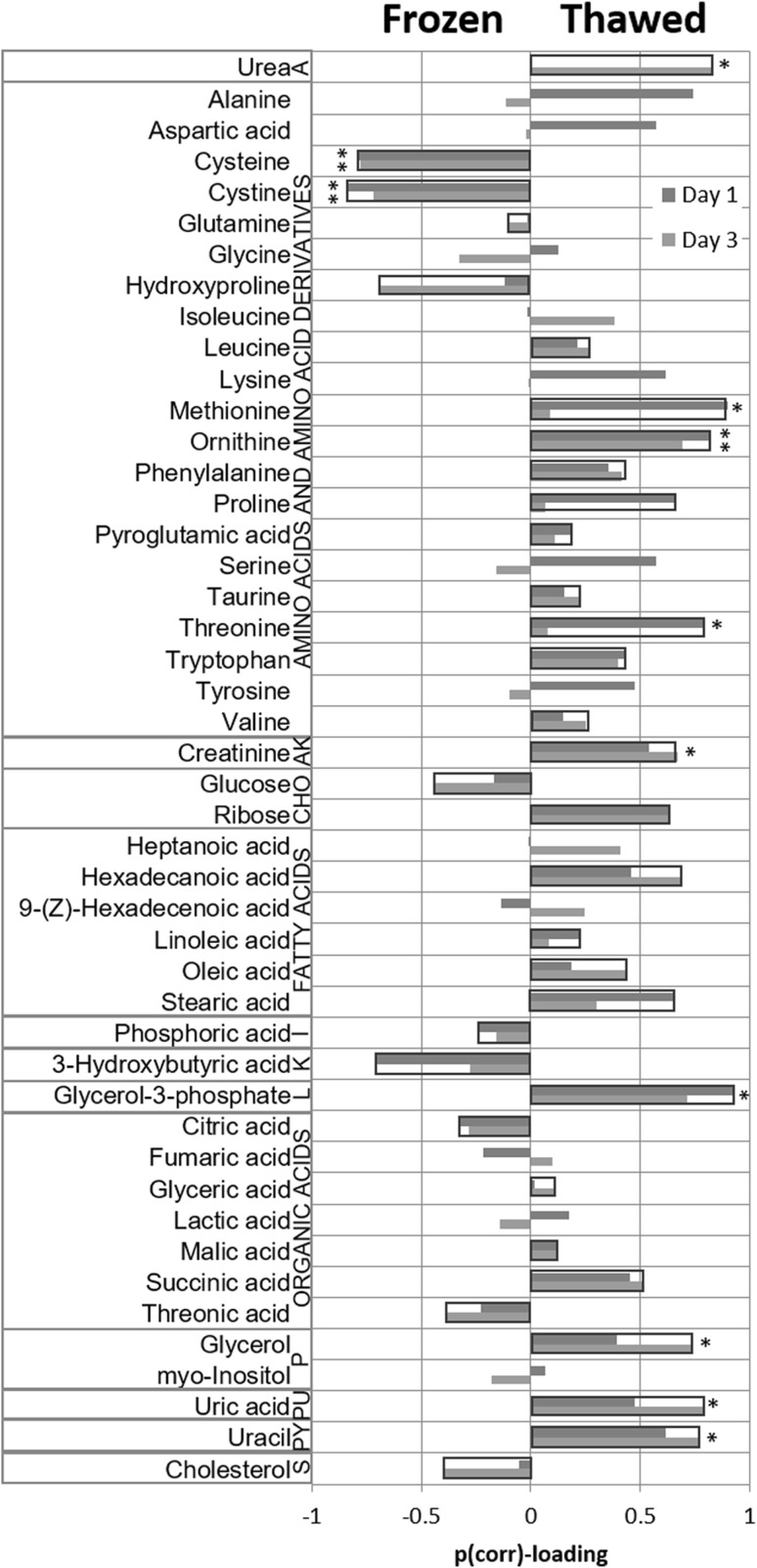
Metabolite differences between frozen and thawed plasma samples. The metabolites in the left-hand column were found at higher levels in the frozen samples whereas the metabolites in the right-hand column (with a positive p(corr)-loading value) were higher in the thawed samples. *Asterisks* indicated the number of days that the difference between frozen and thawed were statistically significant (one *asterisk* represent that the difference was statistically significant one day, two *asterisk* showed that the difference between frozen and thawed samples was statistically significant on both days). Abbreviations used in the legend: *A* amine, *AA* amino acid, *AK* amino ketone, *CHO* carbohydrate, *I* inorganic acid, *K* ketone, *L* lipid constituent, *P* polyol, *PU* purine, *PY* pyrimidine and *S* sterol

When comparing the metabolic pattern of frozen and thawed samples, 34 out of the 46 identified metabolites were found to have the same type of response to thawing, on day 1 and day 3. The statistical significance of these changes were calculated using *t* tests. The p values were then compared to the critical value, calculated using Benjamini–Hochberg FDR method and p values below 0.0087 were found to be significant. Out of the 34 that displayed a similar response on both days, 11 were found to display statistically significant differences between frozen and thawed samples. The p values are available as supplementary data. The differences between frozen and thawed plasma samples from day 1 and day 3 have been summarized in Fig. 2.

Box plots showing the differences, in relative metabolite concentration, between the thawed and frozen samples from day 1, day 3 and day 5 are available as supplementary data.

#### Frozen versus thawed plasma samples from day 5

The samples were obtained by retro-orbital bleeding, on the fifth day of the experiment. In addition, the mice were anesthetized with a ketamine and xylazine solution by intraperitoneal injection. Initially, a two component PCA model was calculated (R^2^X = 0.50). Thereafter, an OPLS-DA model was calculated for the fifth day. The 1 + 1 + 0 component model had R^2^X = 0.45, R^2^Y = 0.89 and Q^2^ = 0.55.

For samples from day 5, two metabolites were found to have a statistically significant difference between the frozen and thawed samples. Samples from day 5 displayed significantly lower concentrations of cysteine (p value = 2.2E−06) and cystine (p value = 1.2E−07). This was also observed in samples from day 1 and day 3. The complete table containing the statistically significant metabolites, is available as supplementary data. The overall metabolic pattern related to thawing was highly similar for samples from all 3 days, even though the number of statistically significant metabolites was lower in samples from day 5. By comparing all 3 days, it was found that the levels of 30 out of the 46 identified metabolites were found to increase or decrease on all 3 days, as a response to thawing.

In order to assess the similarities between the thawing-profiles, from day 1, 3 and 5, Pearson correlation coefficient were calculated. The Pearson correlation coefficient were calculated based on the first p(corr)-loading obtained from the OPLS-DA models. Pearson correlation coefficient for Day 1 and 3 was high (0.60), the same was true for the Pearson correlation coefficient for Day 1 and 5 as well as Day 3 and 5 (0.70 and 0.58, respectively). A figure summarizing the comparisons of the thawed and frozen samples from day 1, 3 and 5 is available as supplementary data.

### Comparison between thawing plasma and organ samples

**Fig. 3:**
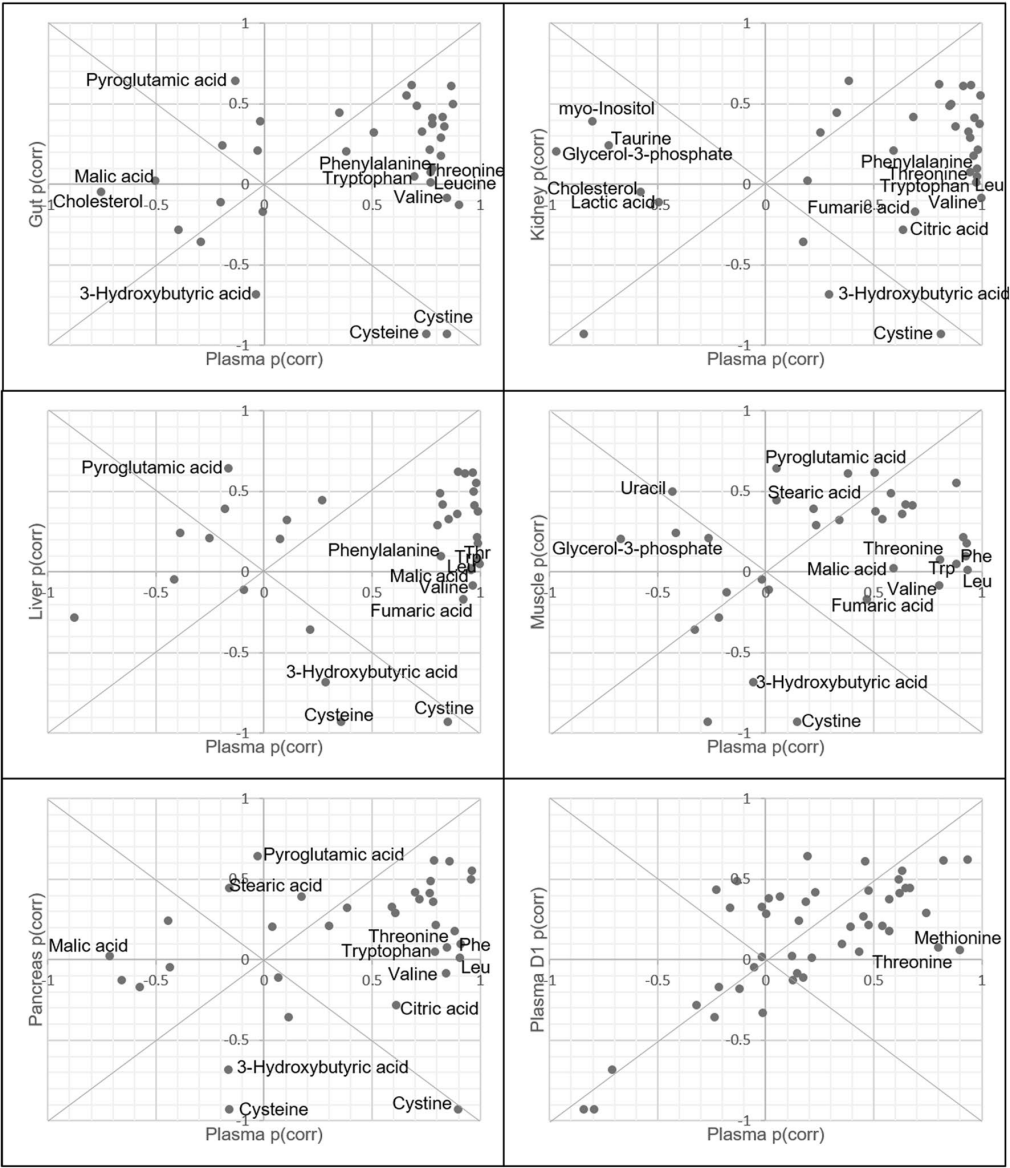
SUS plots comparing plasma day 5 and organs (gut, kidney, liver, muscle and pancreas) and plasma day 1. The p(corr) for plasma was plotted against the p(corr) for each of the investigated organs (gut, kidney, liver and pancreas). This resulted in six separate SUS-plots where the metabolites responsible for the deviating metabolic pattern observed in the thawed plasma samples could be identified. The deviating metabolites have been named in each of the SUS-plots. *Leu* leucine, *Phe* phenylalanine, *Thr* threonine and *Trp* tryptophan

It was decided to use the thawing plasma profile from day 5 and compare it to the previously published organ data (Torell et al. 2015). The samples from day 5 were selected since the organ and plasma samples were harvested on day 5 after the mice had been exposed to anaesthesia (ketamine and xylazine solution). The organs and plasma had 35 identified metabolites in common. The p(corr) values from the OPLS-DA models, calculated to find metabolites discriminating between thawed from frozen samples, were used to create SUS-plots. The SUS-plots were created in order to identify the metabolites that were deviating between the thawed plasma day 5 and the other thawed organs (gut, kidney, liver, muscle and pancreas) as well as plasma day 1, see Fig. 3.

## Discussion

One of the aims of this study was to present a metabolic pattern associated with changes observed in the thawing of plasma samples. This was performed by constructing a type of consensus pattern for samples obtained from three different days. It should be noted that on day 1 and day 3, the blood was drawn from the tail which resulted in a mix of venous and arterial blood whereas the sinus retro-orbital sampling procedure used on day 5 gave only venous blood. This may introduce a difference in metabolic composition between the different sampling days (Hoff and Rlagt 2000). Ketamine has previously been shown to reduce the serum concentration of metabolites involved in glycolysis, TCA cycle, as well as the serum concentration of nucleosides (Overmyer et al. 2015). In the present study, the differences were mainly related to amino acids. However, it is impossible to distinguish the source of the discrepancies as there is a confounding pattern associated with the sampling site as well as anaesthesia. Therefore, the multivariate data analysis of samples from day 5 were performed separately.

The thawed plasma samples were found to be characterized by higher concentrations of creatinine, glycerol, glycerol-3-phosphate, methionine, ornithine, threonine, uracil, urea and uric acid as well as lower concentrations of cysteine and cystine. The samples from day 1 and day 3 displayed stronger discriminations, in the change of metabolite levels, between thawed and frozen samples. Samples from day 5 displayed similar, yet not identical, differences between thawed and frozen samples compared to day 1 and day 3. Differences in response to thawing, between samples from day 1 and day 3 compared to day 5 could have been related to the anaesthesia or sampling site, but it might also have been due to other confounding factors.

In the present study, higher concentrations of most amino acids, fatty acids, purines and pyrimidines as well as metabolites involved in glycerol metabolism, were observed in the thawed samples compared to the frozen ones. This corresponds well to previous studies whereby ornithine, proline, and threonine (Davis et al. 2009; Breier et al. 2014), methionine (Breier et al. 2014), pyroglutamic acid (Hirayama et al. 2015) hexadecanoic acid (C16:0) stearic acid (C18:0) (Eijsden et al. 2005; Breier et al. 2014), urea (Heins et al. 1995), uric acid (Holland et al. 2005; Pinto et al. 2014) as well as creatinine (Clark et al. 2003) have been found in higher concentrations, while cysteine (Hirayama et al. 2015) has been found in lower concentrations, in thawed blood samples. In the present study, metabolites involved in glycerol metabolism (glycerol, glycerol-3-phospahte and glyceric acid) were all observed in higher concentrations in the thawed plasma samples. To the authors’ knowledge, the thawing response of these metabolites have not been studied utilizing a metabolomics approach. The concentration of glycerol and glycerol-3-phosphate was significantly higher, which could potentially be linked to lipolysis (Parasuraman et al. 2010). While low levels of 3-hydroxybutyric acid, could be the result of the decreased fatty acid oxidation (Gomez-Cabrero et al. 2014). The increased concentration of the majority of amino acids implied that there was ongoing protein degradation, e.g. enzymatic catabolism, in the thawed samples. The increased fatty acid concentration was a clear sign of cell membrane degradation and triaglycerol degradation that occur after repeated freeze/thaw cycles (Steponkus and Lynch 1989).

The major goal with this study was to make a comparative study between the obtained consensus plasma profile and the previously published thawing effects on multi-organ samples (Torell et al. 2015), in order to evaluate the differences and similarities between plasma and organ samples from gut, kidney, liver, muscle and pancreas, in response to thawing.

The Pearson correlation coefficients indicated that the difference between frozen and thawed plasma samples were highly conserved, independent of different sampling days. Even though the differences between thawed and frozen plasma samples were much weaker (i.e. displayed fewer statistically significant differences between thawed and frozen samples and with a lower Q^2^ value for the OPLS-DA model for thawed versus frozen samples) in day 5, they still displayed similar characteristics. Samples from day 5 were obtained after the mice had been anesthetized with a ketamine and xylazine solution by intraperitoneal injection. This was performed in order to harvest the organs. Therefore, the samples from day 5 were used for the comparison between thawed plasma and organs.

Out of the 35 common metabolites identified in both plasma and organs, 13 were found to vary in a similar pattern in thawed plasma from day 5 and all thawed organ samples. The similarities between thawed plasma and all of the thawed organ samples included higher concentration of amino acids (alanine, aspartic acid, glutamine, lysine, ornithine, serine and tyrosine), carbohydrates (ribose and glucose) and fatty acids (hexadecanoic acid, 9-(Z)-hexadecenoic acid, linoleic acid and oleic acid). Indicating that the overall trends can be associated with the ongoing protein and cell degradation, observed in the thawed samples from both plasma and organs.

The majority of the differences, associated with the metabolic changes between thawed plasma and thawed organs were fairly similar since the organs’ response to thawing was highly similar, especially with regards to the concentrations of amino acids, carbohydrates and fatty acids (Torell et al. 2015). The SUS-plots revealed that one of the major differences between the thawed organ samples and plasma samples were the concentrations of branched chain amino acids (BCAA; leucine and valine), threonine and aromatic amino acids (AAA; tryptophan and phenylalanine). These metabolites were shown to increase significantly in all of the investigated organs, in response to thawing, while only slightly or no increased could be observed in thawed plasma samples. By comparing the thawed plasma samples from all 3 days, glutamine, glycine, isoleucine, leucine, taurine and valine were found to be stable in the thawed plasma samples. Breier et al. have found glycine and valine to be stable, while they found isoleucine and leucine to increase, during 24 h plasma shipment simulations at RT (Breier et al. 2014). Yang et al. have shown that nine amino acids including BCAAs were stable for 24 h at 37 °C, using rat plasma (Yang et al. 2013). In addition, Davis et al. found glutamine and valine to be stable while they found glycine, isoleucine, leucine and taurine to increase, in whole blood left at RT for 24 h (Davis et al. 2009). The reason for these discrepancies are not fully understood. However, the studies use different sample types as well as different methods for amino acids quantification, introducing several confounding factors. The cystine concentration was lower in the thawed plasma samples, while all of the thawed organs samples displayed higher cystine concentration. The thawed gut and liver samples also displayed high concentrations of cysteine, while the cysteine concentration in pancreas was found not to differ between thawed and frozen samples and was significantly lower in thawed plasma samples. This showed that the proteinogenic amino acids increased to an even greater magnitude in the thawed organ samples than in the thawed plasma samples. One can hypothesize that the availability of enzymes might be one contributing factor or that some of the enzymes involved in the biodegradation of amino acids are more temperature persistent in plasma compared to organ samples. On the other hand, this could be associated with physiological differences between organ samples and blood samples e.g. albumin.

The unique features of plasma in comparison to organ samples comprised of high concentrations of pyroglutamic acid and low concentrations of 3-hydroxybutyric acid. The concentrations of pyroglutamic acid were shown not to change as a response to thawing, in the majority of the organ samples, with the exception of the thawed kidney samples, where the pyroglutamic acid concentration was high. All of the organ samples displayed negligible alterations in 3-hydroxybutyric acid concentration, as a response to thawing. The reason for the discrepancies between thawed plasma and organs are not yet fully understood, but might be related to densities of the samples as well as enzyme availability and sample contents.

TCA cycle intermediates (citric acid, fumaric acid and malic acid) were found to be stable in the thawed plasma samples, from all 3 days. The concentration of citric acid was found to be higher in thawed kidney and pancreas samples. The concentration of fumaric acid was strongly increased in the thawed kidney, liver and muscle samples as a response to thawing. While the concentration of malic acid was found to be higher in thawed liver and muscle samples, but also lower in thawed gut and pancreas samples. The biochemical reactions that constitute the TCA cycle are steps in the cellular energy metabolism. The concentration of the various TCA intermediates were very variable both within sample type, on an individual level, and between sample types on an organ level. One reason may be that TCA intermediates are highly interchangeable and also work as inhibitors for enzymes interconverting them. High concentrations of TCA intermediates can potentially be explained by the fact that the TCA cycle intermediates have a tendency to leak out of cells with a damaged cell membrane (Sellick et al. 2011). Low concentrations on the other hand, may be explained by the utilization of TCA cycle intermediates in biochemical processes associated with the ongoing degradation.

Glycerol-3-phosphate was found in higher concentrations in the thawed plasma samples, while the thawed kidney and muscle samples displayed markedly lower concentrations of this metabolite. The reason could be that triaglycerols were hydrolysed in the thawed plasma samples, resulting in increased levels of fatty acid and glycerol-3-phosphate. The majority of the fatty acids were found to decrease in response to thawing, in both plasma and the investigated organs, with the exception of the stearic acid concentration in muscle and pancreas, which was not affected by thawing.

The thawed organ samples, especially gut and kidney, were characterized by declining cholesterol concentrations, while the thawed plasma samples displayed no significant alteration in cholesterol concentrations, in response to thawing. This indicated that lipid transfer or structural changes of lipoproteins occurred during thawing of the organ samples. The importance of reliable measurements of plasma cholesterol is evident since it is widely used for clinical purposes. The present study showed that cholesterol is fairly stable in plasma, but not in organ samples. The stability of cholesterol in blood samples has been debated, contradictory results have been found when investigating the effect of freeze–thaw cycles on the cholesterol concentration. The cholesterol concentration has been found to increase (Cuhadar et al. 2013), decrease (Zivkovic et al. 2009) and remain stable (Comstock et al. 2008; Kronenberg et al. 1994).

Several of the differences in response to thawing, were unique for individual organ types. For example, the SUS plot for plasma and kidney revealed that the kidney samples responded to thawing with decreased concentrations of lactic acid, while the concentration of lactic acid in plasma did not differ between thawed and frozen samples. This could potentially be explained by the fact that the kidneys and the liver are the major organs involved in lactate metabolism (Bellomo 2002). Indicating that the thawed kidney samples displayed systematic changes in lactate metabolism. In addition, myo-inositol and taurine concentrations were found to increase in plasma as a response to thawing while the thawed kidney samples displayed decreased concentration of these metabolites. Taurine and myo-inositol function as osmolytes in the kidneys and have been found to co-ordinately respond to aid in cellular protection, which may explain the high concentration of these metabolites (Ruhfus and Kinne 1996). Muscle was the only organ that displayed decreased uracil concentration in response to thawing. The plasma and the remaining organs demonstrated markedly increased uracil concentrations. The reason for the decrease in uracil concentration in muscle is not yet fully understood.

## Conclusions

The main aim of this study was to investigate the effects of thawing on the metabolic profiles of different sample types (i.e. gut, kidney, liver, muscle, pancreas and plasma). The comparison between thawed organ and plasma samples indicated that the organ samples were much more sensitive to thawing. The majority of identified metabolites were altered in response to thawing in all five organ groups. These included amino acids, carbohydrates, fatty acids, lipids and nucleic acids. Cholic acid, galacturonic acid, 3-hydroxybutyric acid, phosphoric acid and pyroglutamic acid were the only metabolites found to be stable in the organ tissues. Discrepancies between organs may have been attributed to differences in cellular protection, biocomposition and enzymatic availability.

Although the metabolic profile of plasma was also altered dramatically in response to thawing (with increased levels of amino acids, fatty acids, glycerol metabolites and purine and pyrimidine metabolites), a number of metabolites were found to remain at stable levels. These included several amino acids (glutamine, glycine, isoleucine, leucine, taurine and valine) and TCA cycle intermediates (citric acid, fumaric acid, lactic acid and malic acid). In certain circumstances, plasma samples must be thawed, aliquoted and distributed to multiple sites for different analyses. In these cases the use of repeatedly thawed plasma may be unavoidable. Although caution should be taken when using these samples for metabolic profiling studies, in this study, we have shown that the use of thawed plasma can be acceptable for the measurement of specific metabolites, which remain stable in response to thawing.

## Electronic supplementary material

Below is the link to the electronic supplementary material.

Supplementary material 1 (DOCX 71 KB)

Supplementary material 2 (DOCX 1652 KB)

Supplementary material 3 (DOCX 343 KB)

Supplementary material 4 (DOCX 13 KB)

Supplementary material 5 (DOCX 15 KB)
