# Supplementary material for: The effects of thawing on the plasma metabolome: evaluating differences between thawed plasma and multi-organ samples
Source: Metabolomics. 2017 Apr 17;13(6):66. doi: 10.1007/s11306-017-1196-9 (PMC5392536; doi:10.1007/s11306-017-1196-9)
Supplement: Supplementary file 1 — Supplementary material 1 (DOCX 71 KB) [file 11306_2017_1196_MOESM1_ESM.docx]

**
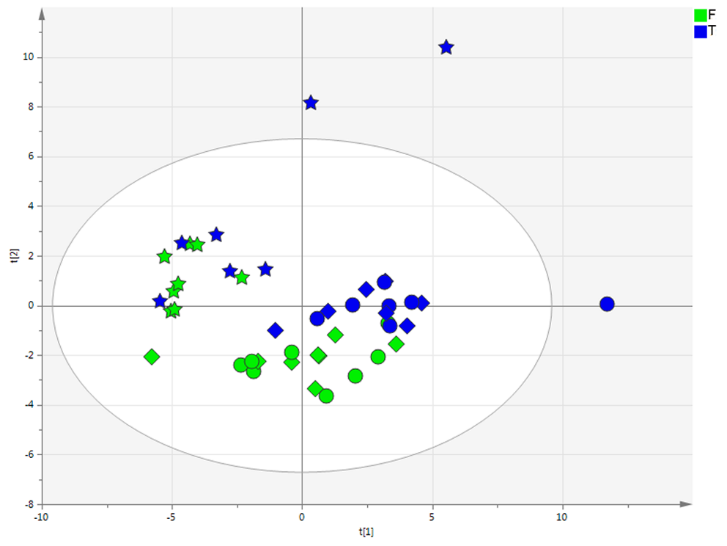
**

**Figure S1: PCA score plot.** PCA score plot showing both the frozen and the thawed samples from day 1, 3, and 5. The two components explained 29 % and 14 % of the variation, respectively. There was no separation between frozen and thawed samples in the first component. The first and second component mainly represent the effects of the anesthesia. But the second component also includes the separation between thawed and frozen samples. The blue spots represent the thawed samples (T) and the green spots the frozen samples (F). Samples from day 1 are represented by circles, samples from day 3 are represented by diamonds and samples from day 5 are represented by stars.
