# Supplementary material for: The effects of thawing on the plasma metabolome: evaluating differences between thawed plasma and multi-organ samples
Source: Metabolomics. 2017 Apr 17;13(6):66. doi: 10.1007/s11306-017-1196-9 (PMC5392536; doi:10.1007/s11306-017-1196-9)
Supplement: Supplementary file 2 — Supplementary material 2 (DOCX 1652 KB) [file 11306_2017_1196_MOESM2_ESM.docx]

**
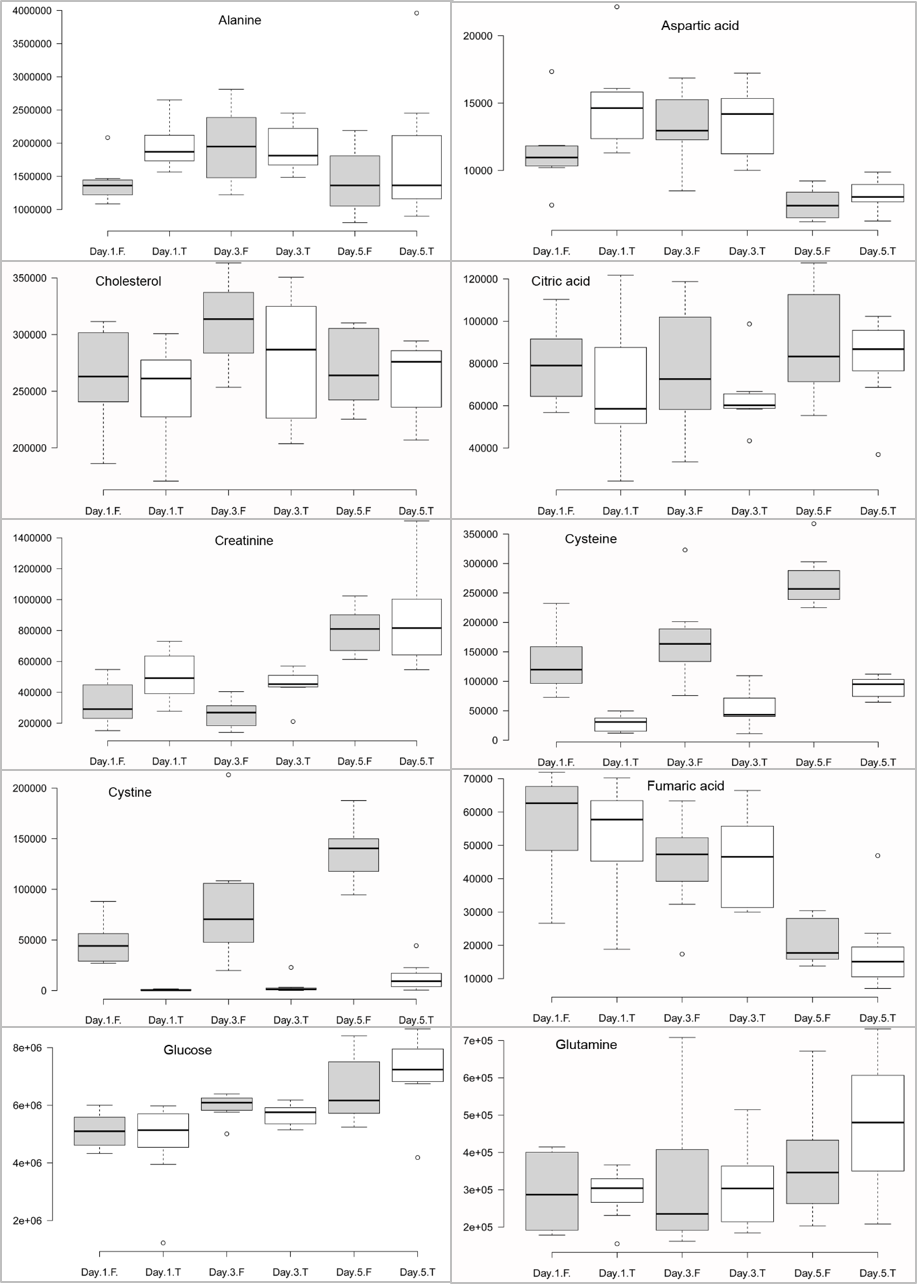
**

**
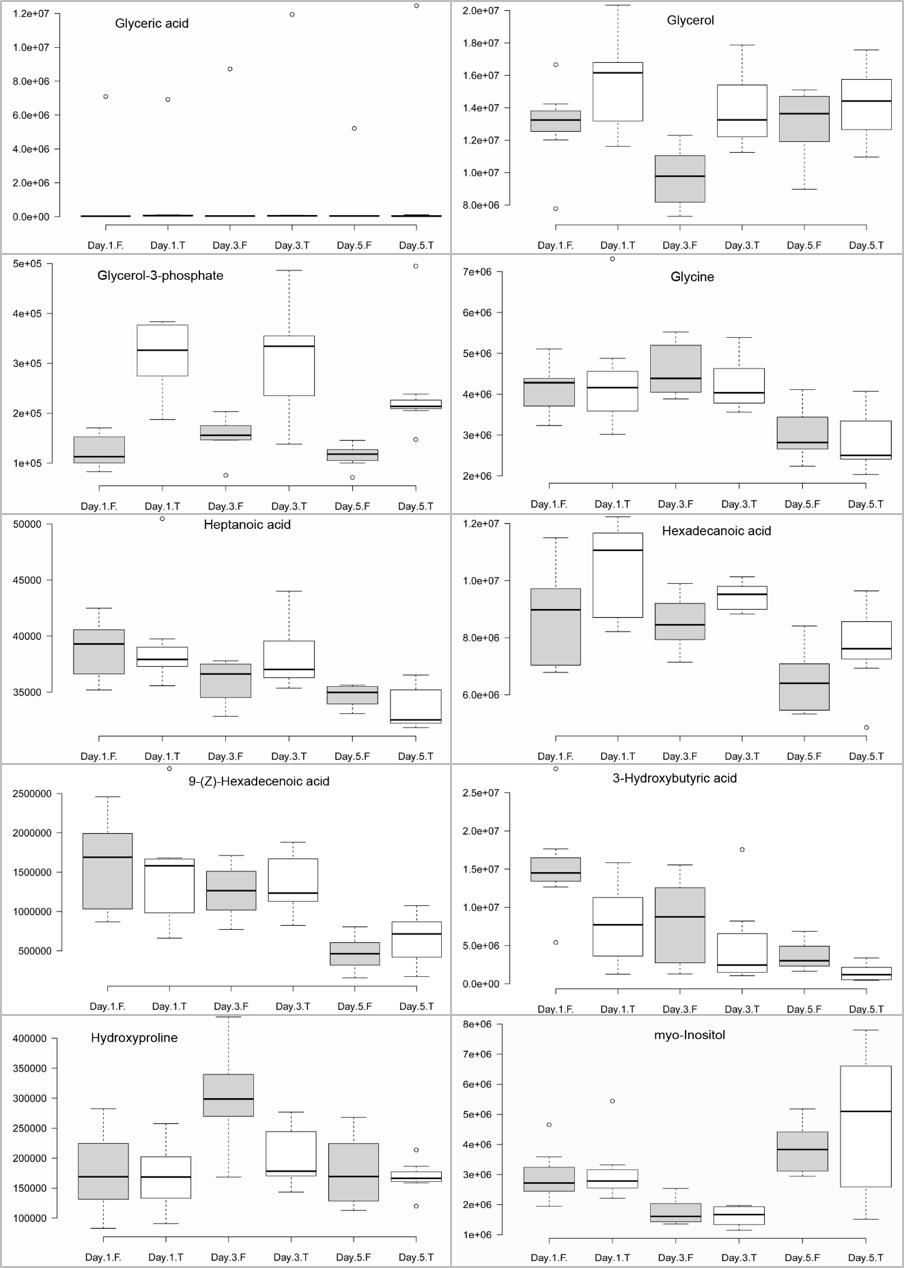
**

**
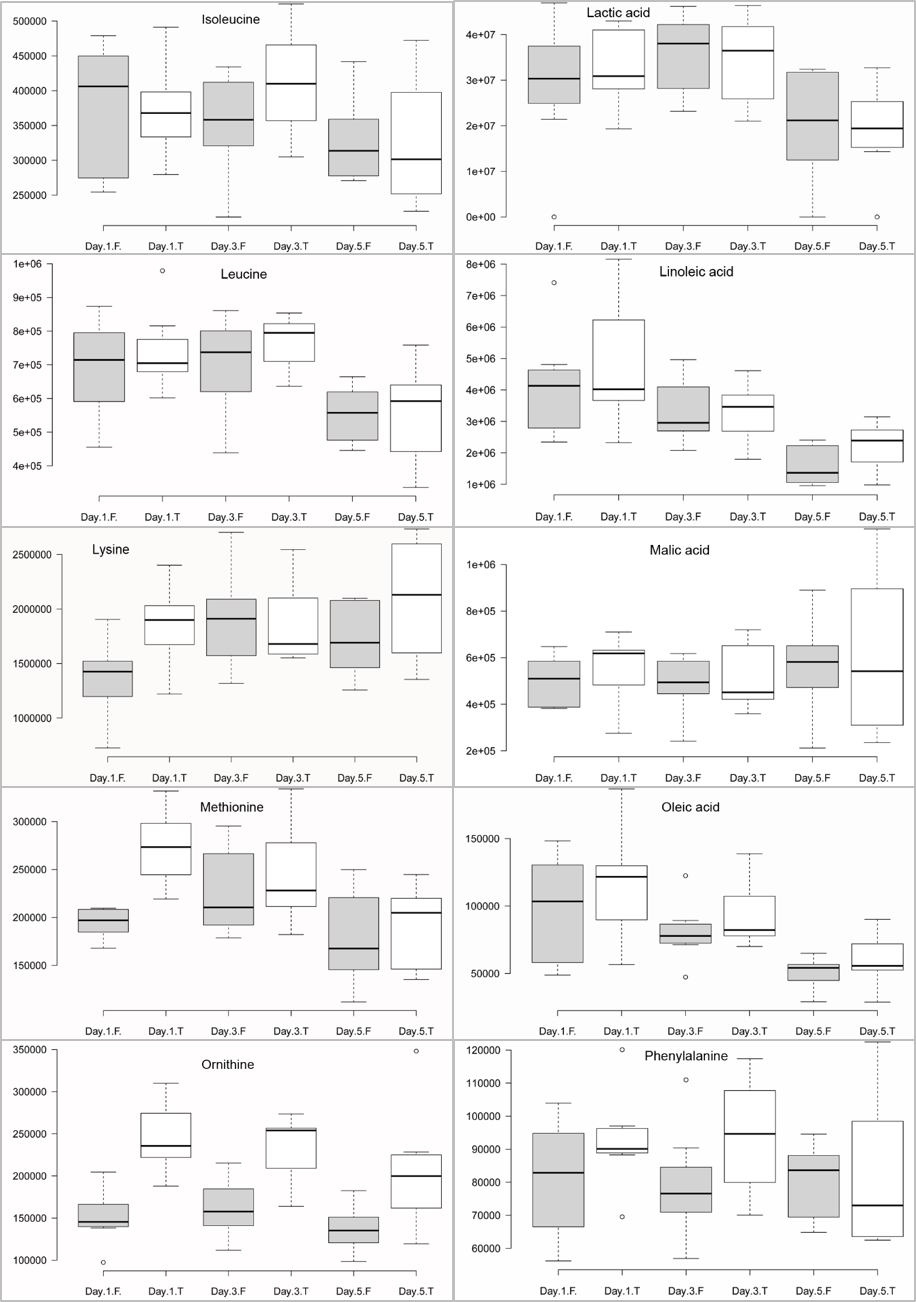
**

**
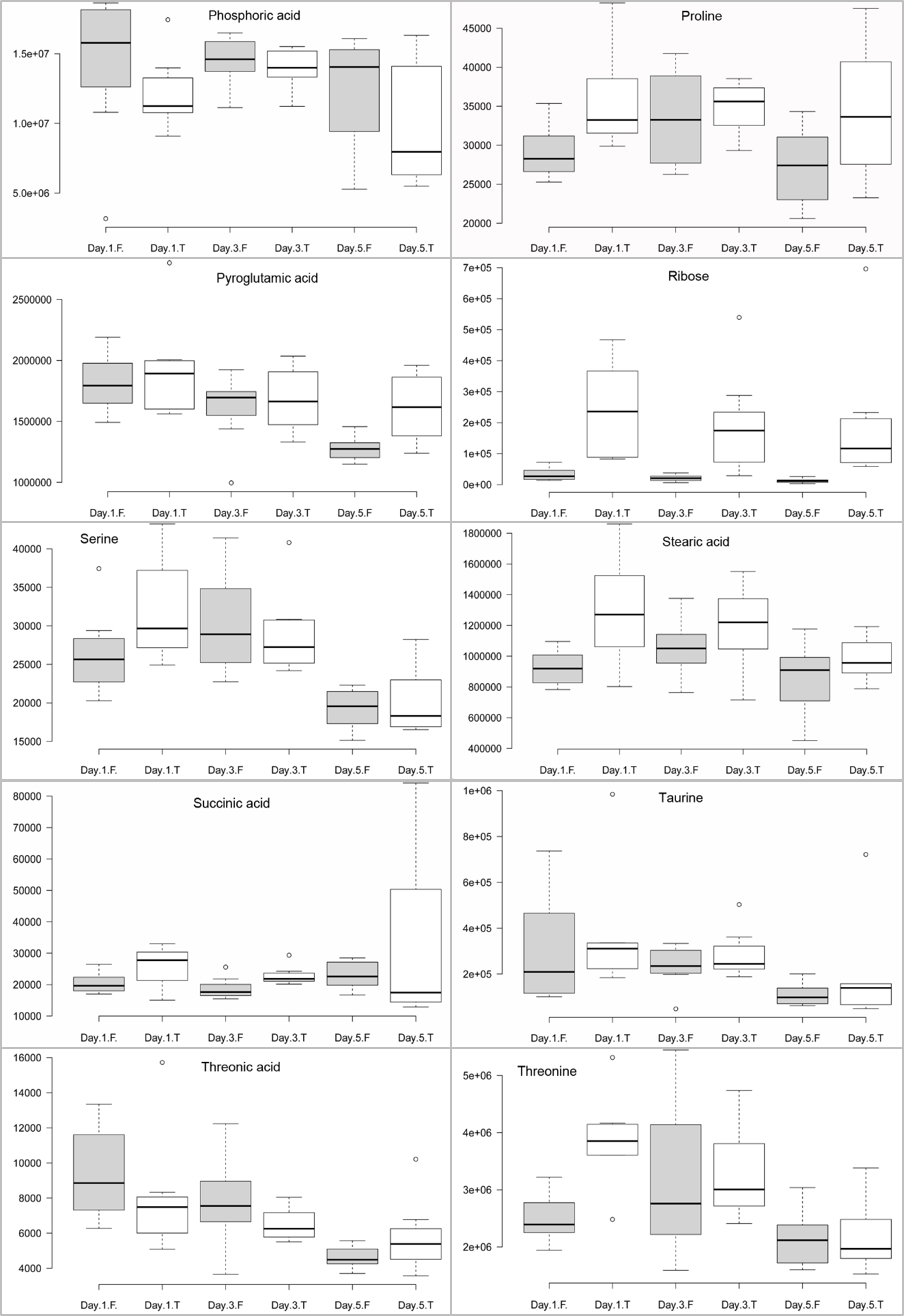
**

**
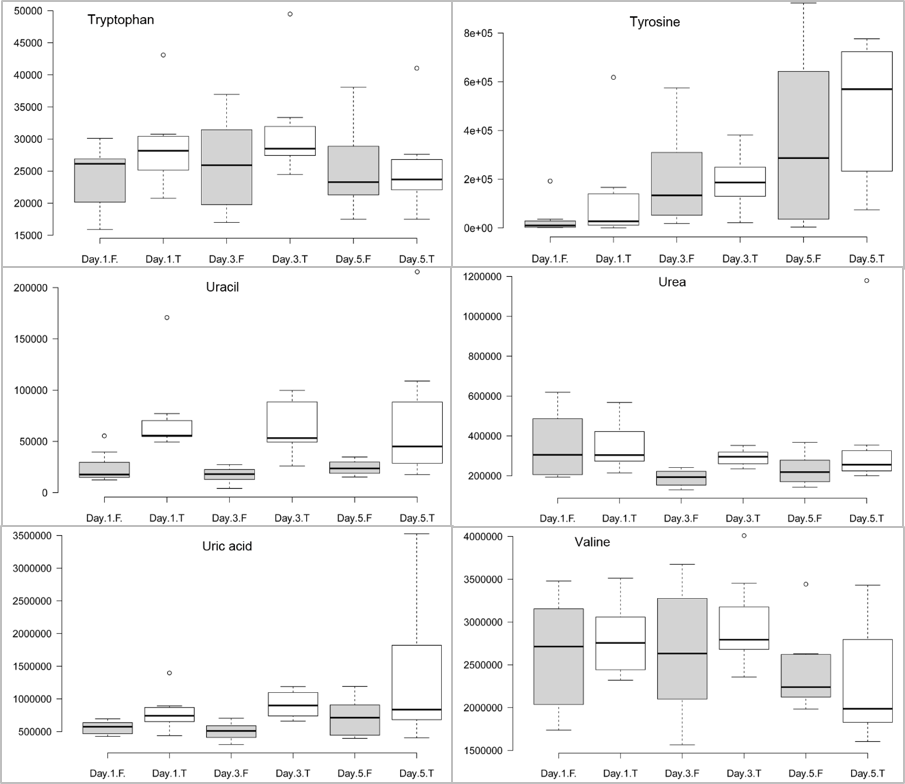
**

**Figure S2: Box-plots.** Box-plots showing the relative concentration in the plasma samples from the thawed and frozen samples from day 1, day 3 and day 5. The frozen samples are represented by grey boxes, while white boxes represent the thawed samples. The metabolites are sorted alphabetically. Centre lines represent the group medians. The box limits indicate the 25th and 75th percentiles as determined by R software. The whiskers are Tukey whiskers, and hence they are extend 1.5 times the interquartile range from the 25th and 75th percentiles. The dots represent outliers. The frozen samples (n = 8 x 3) are represented by grey boxes, while white boxes represent the thawed samples (n = 7 x 3). The metabolites are sorted alphabetically.
