# Supplementary material for: The effects of thawing on the plasma metabolome: evaluating differences between thawed plasma and multi-organ samples
Source: Metabolomics. 2017 Apr 17;13(6):66. doi: 10.1007/s11306-017-1196-9 (PMC5392536; doi:10.1007/s11306-017-1196-9)
Supplement: Supplementary file 3 — Supplementary material 3 (DOCX 343 KB) [file 11306_2017_1196_MOESM3_ESM.docx]

**
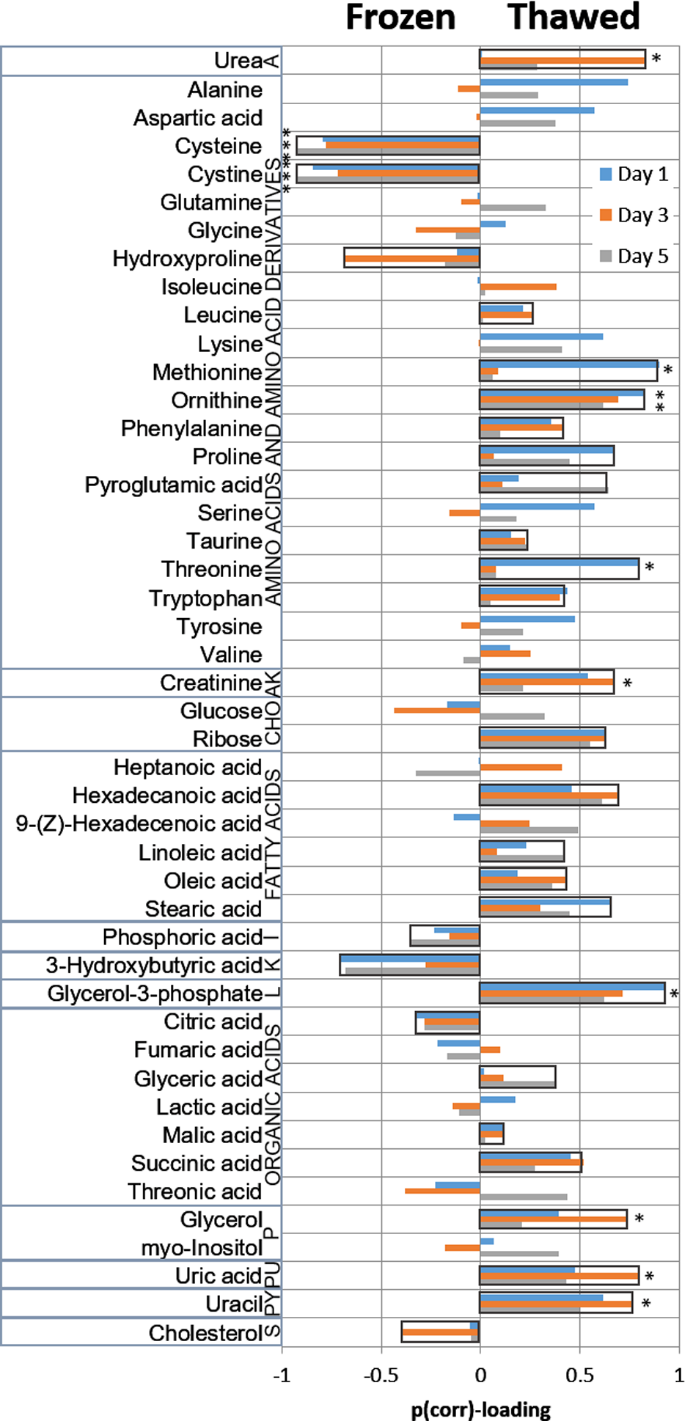
**

**Figure S3: Differences between thawed and frozen plasma.** The metabolites to the left in the picture are higher in the frozen samples while the metabolites to the (with a positive p(corr)-loading value) were higher in the thawed samples. Asterisks indicated the number of days that the difference between frozen and thawed were statistically significant (one asterisk represent that the difference was statistically significant one day, three that the difference between frozen and thawed samples was statistically significant for the samples from all three days). Abbreviations used in the legends: A = amine, AA = amino acid, AK = amino ketone, CHO = carbohydrate, I = inorganic acid, K = ketone, L = lipid constituent, P = polyol, PU = purine, PY = pyrimidine and S = sterol.
