# Supplementary material for: The effects of thawing on the plasma metabolome: evaluating differences between thawed plasma and multi-organ samples
Source: Metabolomics. 2017 Apr 17;13(6):66. doi: 10.1007/s11306-017-1196-9 (PMC5392536; doi:10.1007/s11306-017-1196-9)
Supplement: Supplementary file 4 — Supplementary material 4 (DOCX 13 KB) [file 11306_2017_1196_MOESM4_ESM.docx]

**Table S1: Significant metabolites.** The statistically significant differences between frozen and thawed plasma samples. The t-tests were corrected for multiple testing using Benjamini-Hochberg FDR method. The significant p-values (below 0.0087) have been marked in bold.

| **Metabolite** | **Metabolite class** | **Day 1 Frozen vs Thawed** | **Day 3 Frozen vs Thawed** | **Day 5 Frozen vs Thawed** |
| --- | --- | --- | --- | --- |
| Urea | Amine | 0.97 | **0.0004** | 0.28 |
| Cysteine | Amino acid | **0.0005** | **0.002** | **2.2E-06** |
| Cystine | Amino acid | **0.0004** | **0.006** | **1.2E-07** |
| Methionine | Amino acid | **0.002** | 0.45 | 0.70 |
| Ornithine | Amino acid | **0.0002** | **0.003** | 0.052 |
| Threonine | Amino acid | **0.001** | 0.79 | 0.82 |
| Creatinine | Amino ketone | 0.046 | **0.004** | 0.57 |
| Glycerol-3-phosphate | Lipid constituent | **0.0003** | 0.011 | 0.021 |
| Glycerol | Polyol | 0.11 | **0.002** | 0.34 |
| Uric acid | Purine | 0.080 | **0.0006** | 0.19 |
| Uracil | Pyrimidine | 0.020 | **0.003** | 0.11 |
