# Supplementary material for: The effects of thawing on the plasma metabolome: evaluating differences between thawed plasma and multi-organ samples
Source: Metabolomics. 2017 Apr 17;13(6):66. doi: 10.1007/s11306-017-1196-9 (PMC5392536; doi:10.1007/s11306-017-1196-9)
Supplement: Supplementary file 5 — Supplementary material 5 (DOCX 15 KB) [file 11306_2017_1196_MOESM5_ESM.docx]

Text S1: Materials and Methods

S.1. Materials

All standard reagents were of analytical grade or equivalent and obtained from Sigma-Aldrich (St Louis, MO, USA), Merck (Darmstadt, Germany) and J.T. Baker (Phillipsburg, NJ, USA). N-Methyl-N-trimethylsilyltrifluoroacetamide (MSTFA) plus 1 % trimethylchlorosilane (TMCS) and pyridine were obtained from Thermo Fisher Scientific (Rockford, IL, USA). The stable isotope-labelled internal standards, [^13^C_5_]-proline, [^2^H_4_]-succinic acid, [^13^C_5_,^15^N]-glutamic acid, [1,2,3-^13^C_3_]-myristic acid, [^2^H_7_]-cholesterol and [13C4]-disodium α-ketoglutarate were purchased from Cambridge Isotope Laboratories (Andover, MA); [^13^C_6_]-glucose, [^13^C_12_]-sucrose, [^13^C_4_]-hexadecanoic acid and [^2^H_4_]-putrescine were purchased from Campro (Veenendaal, The Netherlands) and [^2^H_6_]-salicylic acid was obtained from Icon (Summit, NJ). Stock solutions of each internal standard were prepared in either Milli-Q water or methanol to a concentration of 0.5 µg/µL.

S.2. Metabolite extraction from plasma

Frozen plasma was thawed at room temperature for 10 min and stored on ice (4 °C). Plasma samples were prepared for GC-TOF-MS analysis by adding 900 µl extraction mix (methanol:H_2_O, 8:2, v/v), containing all eleven isotopically labelled internal standards (7 ng/µl), to 100 µl aliquots of plasma. Each sample was extracted vigorously using a MM 400 Vibration Mill (Retsch GmbH & Co. KG, Haan, Germany) at a frequency of 30 Hz for 3 min, followed by centrifugation at 18 620 g for 15 min at 4 °C. A volume of 200 µl supernatant was transferred to a GC vial and evaporated to dryness in a SpeedVac concentrator (Savant Instrument, Framingdale, NY, USA).

S.3. Derivatisation of samples

A 30 µl sample of methoxyamine (15 µg/µl) in pyridine was added to each GC vial and the resultant mixture was shaken vigorously for 10 min. Methoxymation was performed at room temperature for 16 h, followed by the addition of 30 µl MSTFA with 1% TMCS to each sample (brief vortex). Samples were left at room temperature for 1 h to allow silylation to occur, followed by the addition of 30 µl heptane (containing 15 ng/µl methyl stearate as an internal standard) and a brief vortex for 10 s. For selected samples technical replicates were also performed, where the whole extraction process was repeated (in triplicate) on the same plasma sample. For these triplicates, the mean values were used.

S.4. GC-TOF-MS analysis

A volume of 1 µl of each derivatised sample was injected splitless by a CTC Combi Pal autosampler (CTC Analytics AG, Zwingen, Switzerland) into an Agilent 6980 GC equipped with a 10 m X 0.18 mm i.d. fused-silica capillary column chemically bonded with 0.18-um DB 5-MS stationary phase (J&W Scientific Folsom, CA). The injector temperature was set to 270 °C. Helium was used as the carrier gas at a constant flow rate of 1 mL min-1 through the column. For every analysis, the purge time was set to 60 s at a purge flow rate of 20 mL min-1 and an equilibrium time of 1 min. The column temperature was held initially at 70 °C for 2 min, then increased to 320 °C at a rate of 30 °C min^-1^, where it was held for 2 min. The column effluent was introduced into the ion source of a Pegasus III time-of-flight mass spectrometer (Leco Corp., St Joseph, MI). The ion source and transfer line temperatures were set to 200 °C and 250 °C, respectively. Ions were generated by a 70-ev electron beam at a current of 2.0 mA. Masses were acquired in the mass range 50-800 m/z at a rate of 30 spectra s^-1^. The acceleration voltage was turned on after a solvent delay of 150 s. The detector voltage was 1670 V.

S.5. Data analysis of all samples analysed by GC/MS

Nonprocessed MS files from GC/TOF-MS analysis were exported in NetCDF format to MATLAB software 7.11 (Mathworks, Natick, MA), where all data pretreatment procedures, such as baseline correction, chromatogram alignment, time-window setting and multivariate curve resolution (MCR) [32] were performed using custom scripts. Automatic peak detection and mass spectrum deconvolution with ChromaTof software were performed using a peak width set to 2 s.

For the identification of metabolites, NIST MS Search 2.0 software was used to compare the mass spectra of all detected compounds with spectra in the NIST library 2.0, the in-house mass spectra library established by Umeå Plant Science Centre and the mass spectra library maintained by the Max Planck Institute in Golm (http://csbdb.mpimp-golm.mpg.de/csbdb/gmd/gmd.html). A retention index comparison was performed, with a retention index deviation <+-10 (in addition to a high spectral match) resulting in a positive ID. LECO ChromaTOF software v4.32 (Leco Corp., St Joseph, MI) was also used as an additional tool for metabolite identification.

The data were normalised using all 11 internal standards (eluting over the whole chromatographic time range). To obtain accurate peak areas for the internal standards, 2 unique masses for each compound were specified and the samples were reprocessed using an in-house MATLAB 7.11 based script. A principal component analysis, using these peak areas, was calculated and the T-score value for each sample was used to normalise the resolved data by dividing the peak areas of each sample with the corresponding score value. Multivariate analysis was performed with SIMCA-P+ 12.0.1 software (Umetrics AB, Umeå, Sweden).
